# Co-expression of Foxp3 and Helios facilitates the identification of human T regulatory cells in health and disease

**DOI:** 10.3389/fimmu.2023.1114780

**Published:** 2023-06-07

**Authors:** Lyra Morina, Madalyn E. Jones, Cihan Oguz, Mariana J. Kaplan, Arunakumar Gangaplara, Courtney D. Fitzhugh, Christopher G. Kanakry, Ethan M. Shevach, Maja Buszko

**Affiliations:** ^1^ Laboratory of Immune System Biology, National Institute of Allergy and Infectious Diseases, Bethesda, MD, United States; ^2^ NIAID Collaborative Bioinformatics Resource, National Institute of Allergy and Infectious Diseases, Bethesda, MD, United States; ^3^ Axle Informatics, Bethesda, MD, United States; ^4^ Systemic Autoimmunity Branch, National Institute of Arthritis, Musculoskeletal and Skin Diseases, Bethesda, MD, United States; ^5^ Laboratory of Early Sickle Mortality, National Heart, Lung and Blood Institute, Bethesda, MD, United States; ^6^ Center for Immuno-Oncology, Center for Cancer Research, National Cancer Institute, National Institutes of Health, Bethesda, MD, United States

**Keywords:** regulatory T cell, Foxp3, Helios, SLE, GVHD, SLe

## Abstract

Foxp3 is regarded as the major transcription factor for T regulatory (T_reg_) cells and expression of Foxp3 is used to identify and quantitate Treg cells in mouse models. However, several studies have demonstrated that human CD4^+^ T conventional (T_conv_) cells activated *in vitro* by T cell receptor (TCR) stimulation can express Foxp3. This observation has raised doubt as to the suitability of Foxp3 as a T_reg_ marker in man. Helios, a member of the Ikaros gene family, has been shown to be expressed by 80-90% of human Foxp3^+^ T_reg_ cells and can potentially serve as a marker of human T_reg_. Here, we confirm that Foxp3 expression is readily upregulated by T_conv_ upon TCR stimulation *in vitro*, while Helios expression is not altered. More importantly, we show that Foxp3 expression is not elevated by stimulation of hT_conv_ in a humanized mouse model of graft versus host disease (GVHD) and in patients with a wide variety of acute and chronic inflammatory diseases including sickle cell disease, acute and chronic GVHD, systemic lupus erythematosus, as well as critical COVID-19. In all patients studied, an excellent correlation was observed between the percentage of CD4^+^ T cells expressing Foxp3 and the percentage expressing Helios. Taken together, these studies demonstrate that Foxp3 is not induced upon T_conv_ cell activation *in vivo* and that Foxp3 expression alone can be used to quantitate T_reg_ cells in humans. Nevertheless, the combined use of Foxp3 and Helios expression provides a more reliable approach for the characterization of T_reg_ in humans.

## Introduction

T regulatory cells (T_reg_) maintain immune system homeostasis by suppressing the activation and proliferation of conventional T cells (T_conv_). Foxp3 is a member of the forkhead or winged helix family of transcription factors. A mutation in this gene in humans causes the severe autoimmune condition known as immune dysregulation, polyendocrinopathy, enteropathy, X-linked syndrome (IPEX). Studies have shown that mutations in the Foxp3 gene are linked to lack of suppressive function of CD4^+^CD25^+^ T_reg_. Thus, expression of Foxp3 is required for the development and function of T_reg_ (1, 2). Helios, a member of the Ikaros gene transcription factor family, also plays a major role in the function of T_reg_ (3–5). Thornton et al. (3) demonstrated that ~70-75% of murine Foxp3^+^ T cells co-expressed Helios and that Helios was co-expressed by an even higher percentage (~85-90%) of human Foxp3^+^ T cells. Expression of Helios appears to correlate with the thymic origin of T_reg_ as T_reg_ generated in peripheral sites (pT_reg_) are Helios^-^. However, this conclusion remains controversial.

As Foxp3 may be regarded as a T_reg_ lineage specific transcription factor, expression of Foxp3 should be the ideal marker to identify and quantify T_reg_. Indeed, mice that express fluorescent probes or human cell surface antigens under the control of the Foxp3 promoter have proven to be invaluable tools to isolate T_reg_ for numerous studies. Although some studies suggest that Foxp3 may be transiently expressed by activated murine T_conv_ cells (6), it has been difficult to directly demonstrate this transient expression. Human Foxp3^+^ T_reg_ can be isolated from healthy donors using a combination of cell surface antigen markers (CD25^hi^CD127^lo^) (7, 8). Multiple studies have shown that *in vitro* stimulation of human T_conv_ cells via the T cell receptor (TCR) results in significant (~30%) induction of Foxp3 expression after 3 days of culture. These studies have led to the hypothesis that Foxp3 expression is a general marker of T cell activation during acute or chronic inflammatory responses and not specific to T_reg_ implying that it cannot be used as a definitive marker of T_reg_ *in vivo* (9–12).

The goals of this study are two-fold. First, we wish to determine whether the induction of Foxp3 expression in human T_conv_ cells during autoimmune and inflammatory disease can be documented *in vivo*. Secondly, we will address whether the combined expression of Foxp3 and Helios can be used to more accurately identify human T_reg_. We first demonstrate that Foxp3, but not Helios, expression is significantly upregulated during the activation of human T_conv_ *in vitro*. We then examine the expression of Foxp3 and Helios *in vivo* using the xenogeneic-GVHD (xeno-GVHD) model in which engrafted hPBMCs are activated in NOD-*scid* IL2Rγ^null^ (NSG) mice (13). T cells from patients with 4 distinct inflammatory responses *in vivo* including Sickle Cell Disease (SCD) (14), GVHD, Systemic Lupus Erythematosus (SLE) and critical COVID-19 were then studied to examine Foxp3 and Helios expression to resolve whether these inflammatory environments facilitate the expression of Foxp3 in T_conv_ cells.

The results of our studies indicate a high correlation between Foxp3 and Helios expression in T_reg_. Foxp3^+^Helios^+^ cells uniformly fail to produce IL-2, a major property of T_reg_, while activated CD25^+^Foxp3^-^Helios^-^ cells robustly produce IL-2. Foxp3 expression is not upregulated during either acute or chronic inflammatory responses in humans. This conclusion is based on the observation that when the percentage of Foxp3^+^ cells is increased in disease, the increase is paralleled by a similar increase in Helios^+^ T cells. Collectively, these findings demonstrate that the combined use of both Foxp3 and Helios expression should be used to identify and quantitate T_reg_ in humans and to validate T_reg_ purity in populations used for adoptive T_reg_ immunotherapy.

## Methods

### Mice

NOD.Cg-Prkdc^scid^IL2rg^tm1Wjl^/SzJ (NSG) mice (005557 Jackson Laboratories) were used. All mice were 6–17 weeks old. Animal protocols used in this study were approved by the NIAID Animal Care and Use committee.

### Human peripheral blood mononuclear cells

Healthy human donor buffy coats were obtained from the NIH Clinical Center Blood Bank. PBMCs were obtained from patients treated on Institutional Review Board approved clinical trials with SCD (ClinicalTrials.gov NCT03077542), Allogeneic hematopoietic cell transplant (ClinicalTrials.gov NCT03983850, NCT04959175), and SLE (NIAMS Clinical Protocol 94-AR-0066). All SLE patients fulfilled the revised SLE criteria for the disease. Lupus activity was recorded using the Systemic Lupus Erythematosus Disease Activity 2000 Index (SLEDAI-2K) score. Patients provided written consent to collaborators prior to sample collection. Buffy coats were diluted in PBS and gradually pipetted over Lymphoprep (Stemcell Technologies) and separated via density gradient centrifugation.

### Flow cytometry

Cells (2-3 x 10^6^) were stained. Human BD Fc block (564220) was used to block Fc receptors. A master-mix with surface staining antibodies as well as a live-dead dye was prepared in PBS (Invitrogen Live/Dead Fixable Near-IR Dead Cell Stain Kit). Surface staining master-mix (50 μL) was added to each sample, and they were incubated at room temperature in the dark for 30 mins. Cells were then washed twice with 3% FBS in PBS. Intracellular staining was done overnight at 4°C. Invitrogen™ eBioscience™ Foxp3/Transcription Factor Staining Buffer Set (Invitrogen, cat 50-112-8857) was used to fix and permeabilize the cells. Prior to acquiring samples, a single stain control was made for each antibody in the flow cytometry panel by incubating 1 drop of UltraComp eBeads (Invitrogen, Cat# 50-112-9040) in 300 μL of PBS and 0.3 μL of each antibody. A Live/Dead staining control was prepared using the ArC™ Amine Reactive Compensation Bead Kit (Invitrogen, Cat# A10346). The compensation matrix was calculated using FACS Diva (**Figure S1**). Cells were acquired using BD Symphony or BD Fortessa. Around 1 million events were acquired per sample. When a new experiment was being conducted, isotype controls and Fluorescence-Minus-One (FMO) controls of cells were prepared to assist with gating. Once acquired samples were analyzed on FlowJo, distribution of events was compared to those of isotype and FMO controls to confirm proper compensation and gating. For some experiments, the compensation matrix was adjusted according to the control samples on FlowJo. All experiments were gated on lymphocytes, single cells, live cells, and CD3^+^ cells to obtain CD4 and CD8 T cell populations.

### Antibodies used for flow cytometry

Anti-human CD45 (HI30), anti-mouse/human Helios (22F6) were obtained from BioLegend. Anti-Human CD127 (HIL-7R-M21), anti-Human CD3 (UCHT1), anti-Human CD4 (SK3), anti-Human TNFα (Mab11), anti-Human CD25 (M-A251), anti-Human IFN-γ (B27), anti-Human Granzyme B (GB11), anti-Human IL-2 (MQ1-17H12), anti-Human CD25 (2A3) anti-Human Foxp3 (236A/E7) were obtained from BD Horizon. Fluorophores used are indicated in figures.

### T cell activation *in vitro*

PBMCs (2 x10^6^) were plated in 2 mL complete RPMI (RPMI 1640 media with phenol red, 2 mM L-glutamine, 1X Penicillin-Streptomycin, 10% Fetal Bovine Serum, 1 mM HEPES in 0.85% NaCl, 1 mM Sodium pyruvate, 1X non-essential amino acids, 1000X 2-mercaptoethanol in a 12-well plate or 24-well plate. Either aCD3/aCD28 coated beads (Dynabeads™ Human T-Activator CD3/CD28, gibco, Ref: 11132D) at 25 μL/1x10^6^ cells with 30 U of recombinant human IL-2 (TECIN, Hoffman-La Roche Inc.) or soluble anti-CD3 (OKT3, Functional Grade, eBioscience, Cat: 16-0037-81) at 1 μg/mL were added for 3 days. In some experiments, 3 day stimulated cells were restimulated with stimulation cocktail with protein transport inhibitor (PMA + Ionomycin + Golgi Stop; eBioscience Cell Stimulation Cocktail (plus protein transport inhibitors) (500X, Cat: 00-4975-93) for 2 hours to allow for detection of cytokine production.

Xeno-mixed leukocyte reaction (MLR) studies were performed by culturing hPBMCs (0.5x10^6^) with spleen cells (0.5x10^6^) from NSG mice. On day 3, 5, and 7, the cells were stained and analyzed via flow cytometry.

### *In vivo* activation of hPBMC in NSG mice

hPBMCs (30x10^6^/mouse) from 3 donors were injected retro-orbitally into NSG mice that had previously been irradiated (150 rad). On day 5, 11, 14, 19, and 27, the spleens were harvested and processed to obtain a single cell suspension. Cell surface and intracellular staining was performed as described above. FACS plots are gated on live, singlet, lymphocytes that are human CD45^+^CD3^+^CD4^+^ T cells. Some mice received intraperitoneal injections of rhIL-2 on 3 consecutive days starting on day 15. On day 18, spleens were harvested from all mice and processed to obtain a single cell suspension. Cells were stimulated for 2 hours with stimulation cocktail, stained, and analyzed via flow cytometry.

### Analysis of CITE-seq data set

Single-cell RNA seq data of PBMCs generated from patients infected with COVID-19 and age and sex matched healthy controls was analyzed to observe whether cells expressing the genes FOXP3, IKZF2 (encoding Helios), and IL2RA display a T_reg_ or T effector cell signature. In the original study by Liu et al. (15), CITE-seq was used to profile the PBMCs to measure the expression of 188 surface markers, the B and T cell receptor sequences of the V(D)J region, and the mRNA transcriptome. The cells with the T_reg_ and T effector signatures to be analyzed were isolated using clustering, manual annotation, and the gating strategy described in Liu et al. (15). Differential expression analysis across the four cell-type patient group subpopulations was performed as well. Visualization of the single cell RNA-Seq data and clustering was performed using the Seurat v4.1.0 package (16) in R with the functions including FindClusters, DimPlot, DotPlot, FeaturePlot, and RunUMAP that utilizes the non-linear dimensionality reduction algorithm called uniform Manifold Approximation and Projection (UMAP) (17) whereas differential expression analysis was performed with MAST (18), or “Model-based Analysis of Single-cell Transcriptomics”. A T_reg_ signature gene set with 62 genes (19) was extracted as the top ranked predictor genes of the T_reg_ state.

### Statistical analysis

Flow cytometry analysis was performed using FlowJo 10.8.1 software and analyzed for statistical significance with PRISM 9 (GraphPad software). An unpaired Student’s t test was used for single comparisons, while a two-way ANOVA with Tukey’s multiple comparison test was used for multiple comparisons. Differences with p < 0.05 were considered statistically significant. All statistics show mean with standard deviation.

## Results

### Helios as a marker for human Foxp3^+^ T_reg_ cells

**Figure 1:**
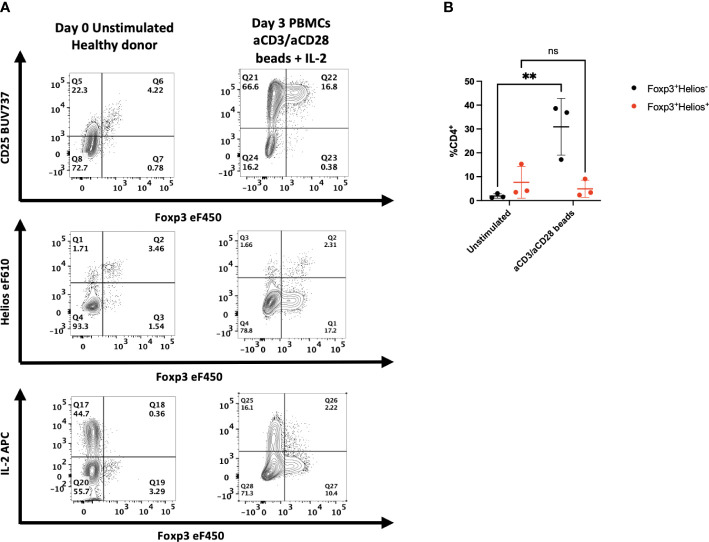
Activation of human PBMC *in vitro* results in upregulation of Foxp3, but not Helios expression. **(A)** PBMCs from healthy donors were left unstimulated or cultured for 72h with anti-CD3/anti-CD28 coated beads and IL-2. All samples were then stained for CD25, Foxp3, Helios or assayed with PMA/ionomycin for 2 hours and stained for IL-2. One representative sample of 3 is shown. Gated on live, CD3^+^, CD4^+^ lymphocytes. **(B)** Cells from stimulated cultures were gated on either Foxp3^+^Helios^+^ or Foxp3^+^Helios^-^ CD4^+^ cells to determine percentages. Results are from three independent experiments. **p<0.005, two-way ANOVA with Tukey’s multiple comparison test.

Our previous studies demonstrated that Helios was expressed by 80-90% of Foxp3^+^ human T cells, and very few CD4^+^Foxp3^-^ T cells (3). As Foxp3 expression has been shown to be upregulated by activation of CD4^+^Foxp3^-^ T cells *in vitro*, we compared the expression of Helios and Foxp3 following *in vitro* activation of human PBMC with anti-CD3/CD28 coated beads and IL-2 (**Figures 1A, B** **)**. In contrast to the marked upregulation of Foxp3 expression, Helios expression was not upregulated by activated CD4^+^Foxp3^-^ T cells. One other property of T_reg_ is their inability to produce effector cytokines. Significant production of IL-2 by Foxp3^-^Helios^-^, but not Foxp3^+^Helios^+^ T cells was observed following *in vitro* stimulation. Activated Foxp3^+^Helios^+^ T_reg_ also failed to produce significant amounts of IFNγ, TNFα, or Granzyme B (**Figure S2**).

It remained possible that the increased percentage of Foxp3^+^ T cells observed after *in vitro* stimulation represented a population of selectively expanded T_reg_ present in the PBMCs that had lost Helios expression. To rule out this possibility, we sorted CD4^+^CD25^-^CD127^hi^ cells, the majority of which are Foxp3^-^, co-cultured them with PBMCs after incubating them in cell trace violet (CTV) and stimulated them with anti-CD3 (**Figure S3**). Following 3 days of stimulation *in vitro*, the majority of the sorted CD4^+^ T cells expressed Foxp3, did not express Helios, and a high percentage produced IL-2 indicating that the Foxp3^+^ T cells generated in culture originated from Foxp3^-^ T cells. These results strongly suggest that co-expression of Foxp3 and Helios may be used to identify human T_reg_ even after *in vitro* stimulation.

It should be noted that 10-40% of CD8^+^ T cells express Helios in freshly isolated healthy donor samples and the percentage of CD8^+^Helios^+^ T cells is highly variable between donors (**Figure S4**).

### Combined use of Foxp3 and Helios to characterize T_reg_ in humanized mice

While Foxp3 expression can be readily upregulated following polyclonal activation of CD4^+^Foxp3^-^ T cells *in vitro*, the upregulation of Foxp3 expression following activation of CD4^+^Foxp3^-^ T cells *in vivo* is unclear. To address this issue, we transferred human PBMCs to NSG mice to induce xeno-GVHD. In parallel, we activated PBMCs from the same donor with NSG splenocytes *in vitro* to mimic the *in vivo* situation (13). As observed, following MLR stimulation *in vitro*, activation by mouse splenocytes induced CD25^+^Foxp3^+^ T cells, the majority of which were Helios^-^ (**Figure S5**).

**Figure 2:**
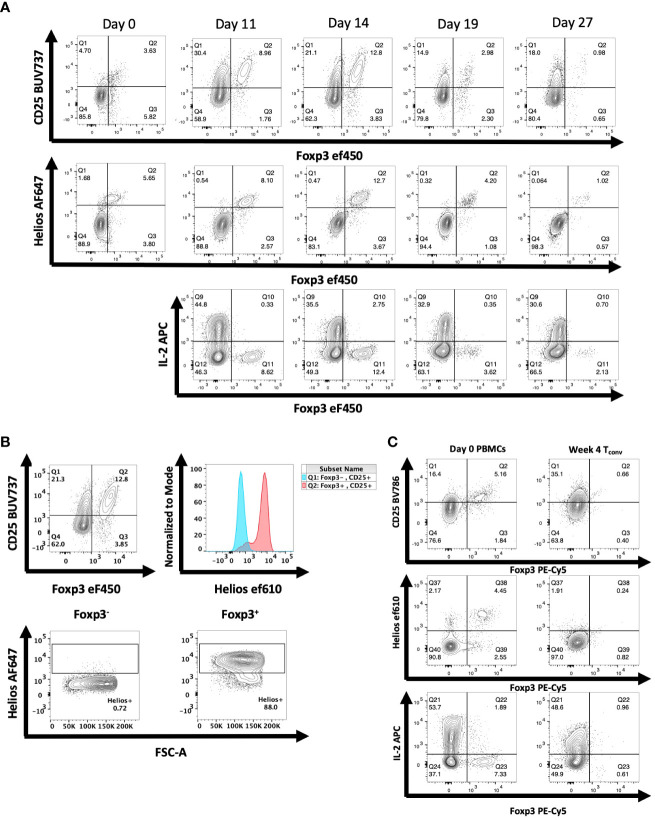
Expression of Foxp3 and Helios in CD4^+^ T cells activated *in vivo* during xeno-GVHD. **(A)** NSG mice received hPBMCs (30 x10^6^) retro-orbitally on day 0 and were analyzed at 4 additional time points. A representative experiment of 3 is shown (*n=19*). Donor cells were stained for CD25, Foxp3, and Helios expression as well as IL-2 production. **(B)** Helios expression was measured in both Foxp3^-^ (blue) and Foxp3^+^ (red) CD4^+^ T cells of a representative mouse shown on day 14 of T cell activation **(C)** NSG mice received CD4^+^CD25^-^CD127^hi^ T cells (3 x 10^6^) retro-orbitally on day 0. On days 21, 28, and 35, spleens were harvested and expression of CD25, Helios, and Foxp3 as well as IL-2 production was analyzed as in **Figure 1**. Data from one representative mouse on day 28 is shown relative to PBMCs from the donor on day 0.

Following transfer of human PBMCs to NSG mice, we observed a time-dependent induction of CD4^+^CD25^+^ T cells (**Figures 2A, B** **)**. The CD4^+^CD25^+^ population could easily be divided into Foxp3^-^ and Foxp3^+^ populations. Maximal expansion of both populations was seen on day 14 after transfer followed by a decline. The CD4^+^CD25^+^Foxp3^-^ cells were Helios^-^ and produced IL-2, while the CD4^+^CD25^+^Foxp3^+^ T cells were Helios^+^ and failed to produce IL-2. Similar results were observed when we transferred sorted CD4^+^CD25^-^CD127^hi^ cells to the NSG mice (**Figure 2C**). Four weeks after transfer, none of the transferred cells expressed Foxp3, approximately 35% expressed CD25, 40% expressed IL-2, and a very low percentage expressed Helios.

### *In vivo* administration of IL-2 does not induce Foxp3 expression by human T_conv_

**Figure 3:**
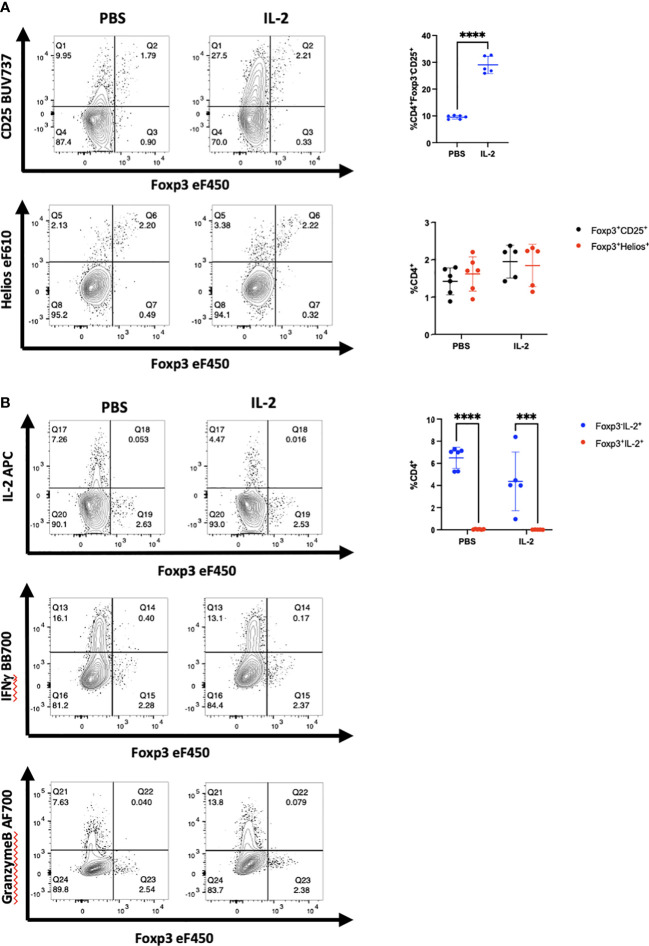
Administration of hIL-2 does not induce Foxp3 expression in T_conv_ during xeno-GVHD. **(A)** NSG mice engrafted with hPBMCs (10 x10^6^) were injected with either hIL-2 (1 x 10^5^ IU, *n=5*) or PBS (*n=*6) on day 11, 12, and 13. CD4^+^ T cells from a representative mouse spleen on day 14 and summary data on CD25, Foxp3 and Helios expression from all recipients are shown. **(B)** IL-2, IFNγ, and granzyme B production by cells in panel A were determined by flow cytometry and summary IL-2 data from all recipients is shown. Plots are representative of one of two independent experiments.***p<0.0005, ****p<0.0001, two-way ANOVA with Tukey’s multiple comparisons test.

A potential difference between the results obtained *in vitro* and those obtained *in vivo* is that the environment for the *in vitro* studies might be regarded as artificial as many of the experiments are performed using polyclonal activation with anti-CD3 coupled to solid surfaces. To test this hypothesis, we enhanced the *in vivo* activation conditions by administering recombinant hIL-2 to NSG mice 11-15 days after PBMC engraftment, a timepoint of peak activation. Following 2 and 3 injections of IL-2, enhancement of CD25 expression was observed compared to PBS injected mice (**Figures 3A**; **S6**). Enhanced CD25 expression was also observed on CD4^+^Foxp3^+^Helios^+^ cells. Nevertheless, most of the cells with enhanced CD25^+^ expression remained Foxp3^-^ and Helios^-^. No enhancement of cytokine production in Foxp3+ cells was observed (**Figure 3B**). We also transferred purified CD4^+^CD25^-^CD127^hi^ T cells to NSG mice and at the peak of their activation *in vivo* administered a single dose of anti-CD3 and analyzed the mouse splenocytes 12h later (**Figure S7**). The activated T_conv_ cells remained Foxp3^-^ and Helios^-^.

### Expression of Foxp3 and Helios during inflammatory and autoimmune disease

While the activation of human T cells in the xeno-GVHD model is a useful model, it does not address potential cellular interactions between the activated T cells and other cell populations that are absent or present at very low frequencies (B cells, dendritic cells, Natural Killer cells, and myeloid cells) in PBMC reconstituted NSG mice and which may play a role in the upregulation of Foxp3 expression by T_conv_ cells. We therefore examined the relationship between Foxp3 and Helios expression in a heterogeneous group of patients with inflammatory and autoimmune diseases.

**Figure 4:**
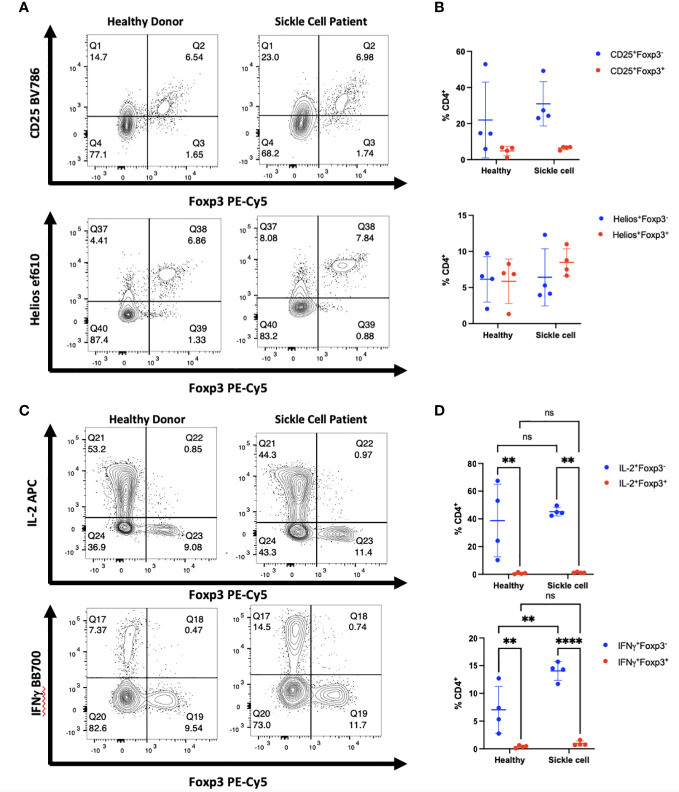
Activated cytokine producing cells in patients with SCD do not express Foxp3. **(A)** PBMCs from patients with SCD and healthy donors were analyzed by flow cytometry. The CD4^+^ population of cells from one representative donor of 4 is shown. **(B)** Summary data on Foxp3 and Helios expression in patients compared with healthy donors (all comparisons are ns). **(C)** PBMCs from one representative patient were analyzed for Foxp3 expression in cytokine producing cells. **(D)** IL-2 and IFNγ production were measured in CD4^+^Foxp3^-^ and CD4^+^Foxp3^+^ groups. **p<0.005, ****p<0.0001, two-way ANOVA with Tukey’s multiple comparison test.

We initially examined a cohort of patients with SCD who have been shown to have elevated levels of T helper cells secreting high levels of IFNγ as well as other inflammatory mediators (14). We obtained PBMCs from patients with severe SCD collected prior to haploidentical hematopoietic cell transplantation (HCT) as well as PBMCs from the healthy donors. Although there was great variability between patients and donors, SCD patients manifested greater activation of CD4^+^ T cells than healthy donors based on high levels of IFNγ production as well as a trend toward higher expression of CD25 (**Figures 4A–D**). However, almost all Foxp3^+^ T cells in the patients expressed Helios and very few of the CD4^+^CD25^+^Foxp3^-^ T cells expressed Helios. Thus, it is unlikely that any of the Foxp3^+^ T cells represent activated effector cells that have upregulated Foxp3. It should also be noted that CD8^+^ T cells in this cohort of patients were also activated and expressed high level of IFNγ and granzyme B compared to healthy controls; none of the activated CD8^+^ T cells expressed Foxp3 (**Figures S8A, B**).

**Figure 5:**
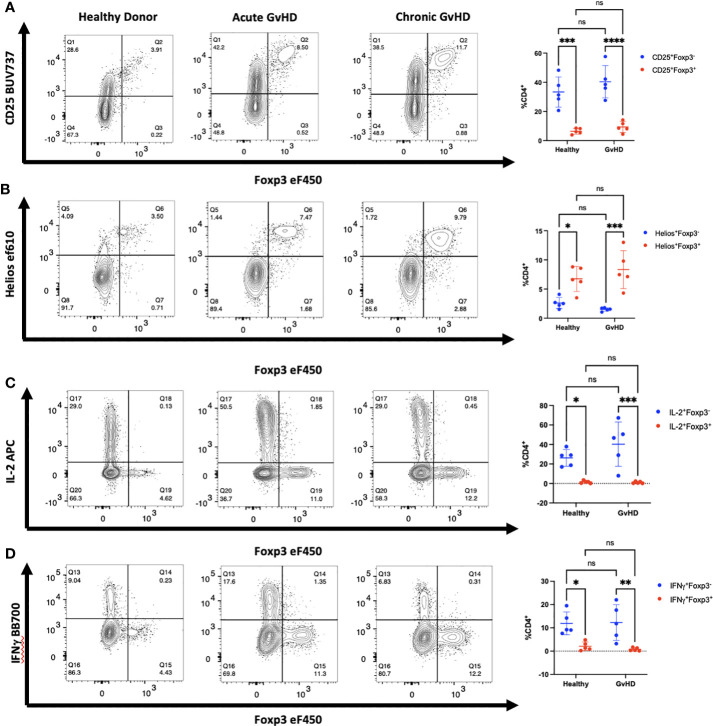
Activated CD4^+^ T cells in patients with GVHD do not express Foxp3. **(A-D)** PBMCs from one representative patient with acute and one representative patient with chronic GVHD obtained at diagnosis as well as one healthy control were analyzed by flow cytometry for expression of CD25, Foxp3, Helios, IL-2, and IFNγ. Summary of data from 5 patients with both acute and chronic GVHD gated on live, CD3^+^, CD4^+^ lymphocytes. *p<0.05, **p<0.005, ***p<0.0005, ****p<0.0001, two-way ANOVA with Tukey’s multiple comparisons test. ns, not significant.

We extended these studies on the potential induction of Foxp3 expression in CD4^+^ non-T_reg_ by examining a cohort of patients who were developing acute GVHD or who manifested signs of chronic GVHD following HCT for malignancies (**Tables 1A**–**C**). We observed a trend in increased percentage of CD25^+^Foxp3^+^ T_reg_ in GVHD patients as compared to healthy donors. In both patient groups, almost all Foxp3^+^ T cells were Helios^+^, while very few Foxp3^-^ T cells expressed Helios (**Figures 5A, B**). In GVHD, CD4^+^Foxp3^-^ T cells demonstrated a trend for enhanced production of IL-2 (**Figure 5C**). No effector cytokine production was observed by Foxp3^+^Helios^+^ T cells suggesting that T_reg_ non-responsiveness was not destabilized under conditions of severe inflammation (**Figure 5D**).

**Figure 6:**
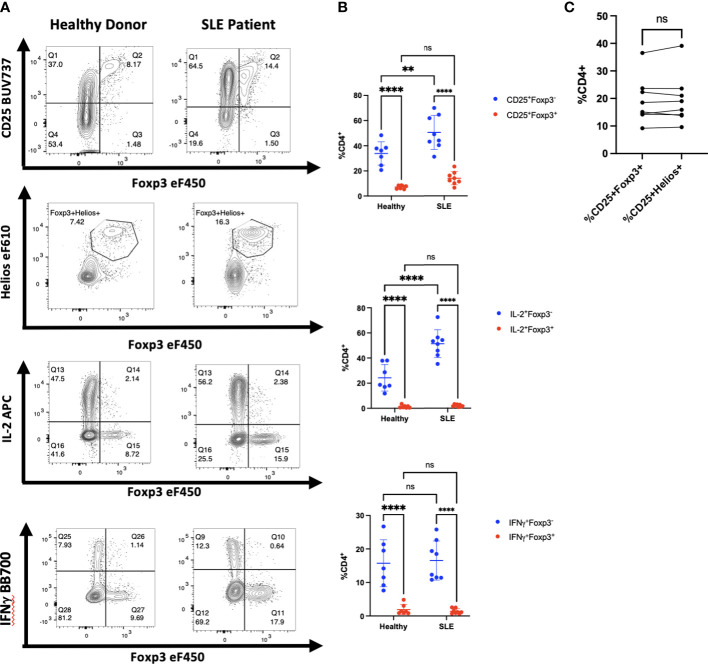
Activated CD4^+^ T cells in patients with SLE do not express Foxp3. **(A)** CD4^+^ T cells from one representative patient with SLE of 8 and one heathy control were analyzed by flow cytometry for expression of CD25, Foxp3, Helios, IL-2, and IFNγ. **(B)** Comparison of CD25 expression, IL-2, and IFNγ production by CD4^+^Foxp3^+^ and CD4^+^Foxp3^-^ T cells from SLE patients and healthy controls. **(C)** Correlation between Foxp3 expression and Helios expression in CD4^+^CD25^+^ T cells from SLE patients. **p<0.005, ***p<0.0005, ****p<0.0001, two-way ANOVA with Tukey’s multiple comparisons test **(B)**, Student’s t-test, ns, not significant **(C)**.

Diminished production of IL-2 by CD4^+^ T cells in patients with SLE has been proposed (20, 21) as a cause for diminished numbers and function of T_reg_. In addition, CD4^+^ T helper activity is reported to be abnormal and overactive in many patients with SLE. To determine whether the increased CD4^+^ T cell activation in SLE would result in upregulation of Foxp3 in non-T_reg_ cells, we isolated PBMCs from whole blood samples obtained from eight patients being treated for SLE. The disease severity, measured using the SLEDAI-2K, had a wide range with scores from 0-10 (**Table 1**). We found that CD4^+^ T cells from most of the patients in our cohort had a higher percentage of CD25^+^Foxp3^-^ T cells than healthy controls and had an upward trend of CD25^+^Foxp3^+^ T cells in comparison to controls (**Figures 6A, B**). Importantly, the percentage of CD25^+^Foxp3^+^ T cells was closely correlated with the percentage of Helios^+^Foxp3^+^ T cells in every patient strongly suggesting that the elevation in the percentages of Foxp3^+^ T cells in the patients was due to expansion of T_reg_ and not to upregulation of Foxp3 expression by non-T_reg_ (**Figure 6C**). We also observed that CD4^+^Foxp3^-^ but not CD4^+^Foxp3^+^ T cells from the patients were responsible for the enhanced production of IL-2 and IFNγ.

**Table 1A:** Clinical characteristics of SCD patients.

Sickle Cell Disease			
Recipient ID	Age	Sex	SCD Type
1	19	Male	HbSS
2	31	Male	HbSS
3	31	Female	HbSS
4	51	Female	HbSS

**Table 1B:** Clinical characteristics of GVHD patients.

Graft-versus-Host Disease						
Patient ID #	Sex	Age	GvHD Type	Transplant Type	Donor Type	Graft Source
1	F	23	Chronic	Reduced intensity conditioning	HLA-haploidentical	Bone marrow
2	F	26	Acute	Reduced intensity conditioning	HLA-haploidentical	Bone marrow
3	F	47	Chronic	Myeloablative conditioning	HLA-haploidentical	Bone marrow
4	M	31	Chronic	Myeloablative conditioning	HLA-haploidentical	Bone marrow
5	M	42	Acute	Reduced intensity conditioning	HLA-matched sibling	Bone marrow

**Table 1C:** Clinical characteristics of SLE patients.

Systemic Lupus Erythematosus						
Patient ID #	Sex	Age	Ethnicity	SLEDAI Score	Disease manifestations	Current medications
1	M	52	African American	2	• +anti-dsDNA	• Hydroxychloroquine • Prednisone • Mycophenolate mofetil • Lisinopril • Rosuvastatin • Metoprolol
2	F	52	Unknown	0	• N/A	• Hydroxychloroquine • Azathioprine • Omeprazole • Acyclovir
3	F	71	Hispanic	0	• N/A	• Prednisone • Azathioprine • Omeprazole • Lisinopril • Metformin • Glipizide
4	F	37	Hispanic	0	• N/A	• Hydroxychloroquine • Prednisone • Azathioprine
5	F	41	Hispanic	6	• Rash • Hypocomplementemia • Increased DNA binding	• Hydroxychloroquine • Prednisone • Azathioprine • Warfarin
6	F	40	Hispanic	4	• Alopecia • Low complement	• Prednisone • Mycophenolate mofetil • Amlodipine • Losartan
7	F	63	African American	2	• Increased DNA binding	• Hydroxychloroquine • Prednisone • Azathioprine • Losartan/HCTZ • Metformin
8	F	63	Hispanic	10	• Arthritis • Alopecia • Low complement • Increased DNA binding	• Hydroxychloroquine • Prednisone • Mycophenolate mofetil • Lisinopril

**Figure 7:**
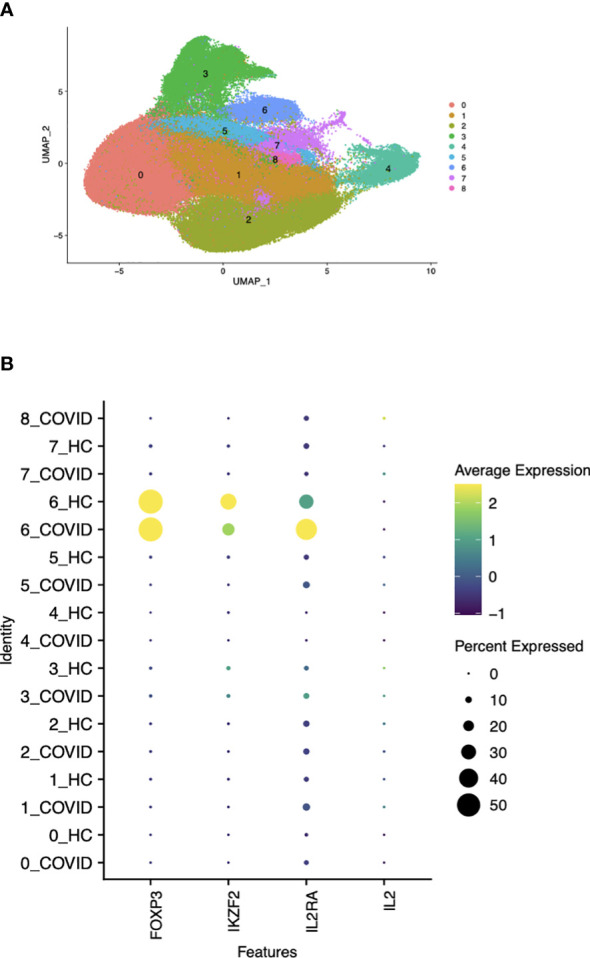
Correlation between Foxp3 and Helios in CD4^+^ T cells by gene expression clustering in patients with moderate to critical COVID-19. **(A)** UMAP visualization of total CD4^+^ T cells and T_reg_ clustering based on protein expression profile labels. **(B)** Detection rates of the genes of interest expressed by cells in either patients with COVID-19 or healthy controls (HC) within each cluster shown in (7A).

An overactive inflammatory response, which causes the release of large amounts of pro-inflammatory cytokines, known as a “cytokine storm,” has been described in patients with moderate to critical conditions of COVID-19 (22). As the inflammatory state in patients with severe COVID-19 likely differs from that seen in the more chronic disease models we have studied, we performed a new analysis on publicly available scRNA-seq data (15). PBMCs from patients with moderate, severe, and critical COVID-19 and age and sex matched healthy controls were profiled in the original study. Our analysis examined whether the expression of FOXP3 was associated with the case control status in the non-T_reg_ subpopulation in any cluster characterized by a high FOXP3 and IKZF2 expression. We re-clustered the CD4^+^ T cells of both patients and healthy controls (cluster 6 in **Figure 7A**) to identify the Foxp3^+^ cell population for follow-up analysis. The remaining clusters were composed of CD4^+^ T cell subtypes including Tregs, naïve CD4^+^ T cells and effector memory cells with no clear separation between them with respect to the cluster identities on the UMAP space. The populations outside cluster 6 were already characterized broadly in Liu et al. and our analysis only focused on this FOXP3^+^IKZF2^+^ cluster. While the expression of these two genes and IL2RA were mainly confined to cluster 6, **Figure 7B** shows that IL2 expression was only detected at a negligible level by the high throughput 10X Genomics single cell assay in any of the clusters. The detection rates (percent of cells expressing a gene) of FOXP3, IKZF2 and IL2RA in clusters other than the T_reg_ cluster (cluster 6) were around 0% and none of these genes were differentially expressed between the two patient groups in T_conv_ (Data not shown). Furthermore, using linear regression, we modeled the FOXP3 expression in cluster 6 as a function of the expression levels of the T_reg_ signature genes from the literature (19), age, gender, batch, and the cell population identity defined by the combined patient status (HC/COVID) and cell type (T_reg_/non-T_reg_). FOXP3 expression was not associated with the non-T_reg_-COVID state (p-value>0.65) indicating that FOXP3 was not abnormally overexpressed in the non-T_reg_ cell population under the conditions of robust inflammation in patients severely ill with COVID-19 (**Table S1**). This observation strongly indicates that Foxp3 was not abnormally upregulated by non-T_reg_ under the conditions of robust inflammation in patients severely ill with COVID-19.

## Discussion

The discovery of T_regs_, a unique, suppressive population characterized by expression of CD25 and Foxp3 with similar phenotype and function in both mouse and humans, generated considerable interest in defining defects in either the number or function of these cells in human autoimmune diseases. The major question that arose at the time was how to best characterize and isolate this unique population. The availability of monoclonal antibodies to Foxp3 that could be used for intracellular staining appeared to provide a major tool for T_reg_ characterization. However, it was rapidly demonstrated that stimulation of human T_conv_ cells in cell culture via TCR signaling induced the expression of Foxp3, although such cells lacked suppressor function (9–12). The *in vitro* findings with human T_conv_ cells raised concerns about the use of Foxp3 as a T_reg_ marker in humans particularly in patients with inflammatory conditions. Several studies (7, 8) demonstrated that the combined use of CD25^hi^ and CD127^lo^ as surrogate markers could facilitate the isolation of highly enriched populations of Foxp3^+^ T cells from normal donors. However, the application of this protocol to define T_reg_ under inflammatory conditions is limited as expression of CD25 is upregulated on all CD4 T cells with TCR activation while expression of CD127 is downregulated. Thus, our ability to accurately define human T_reg_ remains challenging. Other approaches to identify T_reg_ using a combination of cell surface markers (ex: CD4^+^CD25^+^CD226^-^) have only been partially successful (23).

We have made use of expression of the transcription factor Helios together with Foxp3 as an alternative method that accurately identifies most human T_reg_. Helios was originally characterized in the mouse where 70-75% of Foxp3^+^ T cells are also Helios^+^. We originally suggested that thymic T_reg_ (tT_reg_) were Helios^+^ while T_reg_ generated in the periphery (pT_reg_) from T_conv_ cells were Helios^-^ (3). The function of Helios in mouse T_reg_ remains controversial as some studies suggest it is required for survival during the maturation of naïve T_reg_ to effector T_reg_ (4), while other studies suggest it may be involved in responsiveness to IL-2 (24). In human peripheral blood, 85-90% of Foxp3^+^ T cells co-expressed Helios using an anti-mouse Helios monoclonal antibody that cross-reacted with human Helios. Further characterization of human T_reg_ demonstrated that the Foxp3^+^Helios^+^ population had a completely demethylated T_reg_-specific demethylated region (TSDR), while the TSDR of the Foxp3^+^Helios^-^ cells was only 50% demethylated consistent with a pT_reg_ origin (25). Studies of human T_reg_ by Lam et al. (26) following prolonged (>13 days) cell culture *in vitro* under inflammatory conditions have demonstrated that Helios expression is not required for T_reg_ stability, but it remains unclear if these results apply *in vivo*. Nevertheless, Lam et al. (26) agree with the data presented here that measurement of Helios expression in conjunction with Foxp3 is a valuable addition to the characterization of human T_reg_. In addition, the majority of the T_reg_ capable of producing low levels of effector cytokines were found in the Foxp3^+^Helios^-^ population which is also consistent with a less stable T_reg_ phenotype and pT_reg_ origin. We have limited our studies in this report to an analysis of the Foxp3^+^Helios^+^ CD4^+^ T cells.

One critical issue was whether Helios expression could be upregulated in activated human T_conv_ cells similarly to Foxp3. In contrast to mouse T_conv_ cells where significant expression of Helios can be induced by TCR activation of T_conv_ cells *in vivo*, we did not observe Helios expression in human T_conv_ cells after TCR activation following polyclonal activation with anti-CD3 and anti-CD28 or stimulation in a xeno-MLR *in vitro*. A variable percentage of both human and mouse CD8^+^ T cells can express Helios and further studies are needed to determine the significance of Helios expression in CD8^+^ T cells.

One question that remains to be addressed is why human T_conv_ cells upregulate Foxp3 upon activation *in vitro*, while mouse activated T_conv_ cells remain Foxp3^-^. The major factor responsible for induction of Foxp3 expression *in vitro* is TGFβ. We have previously shown that the addition of anti-TGFβ during activation of human T_conv_ cells *in vitro* reduces the upregulation of Foxp3 expression by 90% (11). Thus, it appears that human T_conv_ cells have a greater sensitivity to exogenous TGFβ present in the fetal calf serum used to supplement the culture media. The other difference in the response of human and mouse T_conv_ to TGFβ is that mouse T_conv_ cells induced to expressed Foxp3 in the presence of TGFβ (iT_reg_) have many of the functional properties of tT_regs_ and exhibit suppressor function *in vivo* and *in vitro*, although they do exhibit decreased stability of Foxp3 expression. In contrast, human T_conv_ cells induced to express Foxp3 in the presence of TGFβ, produce IL-2 and lack suppressive function *in vitro*. Further studies are needed to resolve these fundamental differences.

We first validated that the combined use of Foxp3 and Helios expression facilitated the identification of human T_reg_. Polyclonal activation of human T_conv_ resulted in upregulation of Foxp3 expression to levels similar to those detected on unstimulated Foxp3^+^ T_reg_. In contrast, no upregulation of Helios was observed in cultures of purified T_conv_ or unstimulated human PBMCs. Similar results were observed in xeno-MLR cultures. To test whether activation of human T_conv_ resulted in upregulation of Foxp3 expression *in vivo*, we first utilized the xeno-GVHD model. hPBMCs transferred to immunocompromised, NSG mice engraft, but over a period of several weeks recognize mouse-specific antigens, become activated and ultimately induce the death of the recipients secondary to systemic xeno-GVHD. Expansion and activation of T_conv_ as assayed by induction of CD25 expression was routinely observed two weeks after transfer, but no induction of Foxp3 expression was observed even in activated T cells with the highest levels of CD25 expression. Foxp3^+^ T_reg_ also transiently expanded in this model and remained almost 100% Helios^+^.

While the studies in the humanized mice demonstrate a failure of T_conv_ to upregulate Foxp3, this model does not completely mimic activation of human T cells *in vivo* as the only human cells in the NSG mice are the T cells that successfully engraft. Other human cell types or products may be needed to upregulate Foxp3 expression in human T_conv_. We therefore examined T_reg_ in the peripheral blood of patients with manifestations of systemic inflammation including SCD (14), acute and chronic GVHD, and SLE. We observed an elevation in the percentage of T_reg_ in the peripheral blood and an enhanced percentage of CD4^+^Foxp3^-^CD25^+^ T cells. We also examined a single cell RNA-seq analysis of a cohort of patients with acute inflammation secondary to severe or critical COVID-19 (15). Flow cytometry was not performed in this study, but examination of Foxp3 and Helios expression at the molecular levels demonstrated a complete correlation between cells expressing Foxp3 and Helios and other T_reg_ signature genes. Foxp3 and Helios were not expressed in any of the subsets of cytokine producing activated T cells. We recognize that only a small group of patients with each disease were studied and our analysis was limited to PBMCs. It remains possible that patients with a different clinical course or with other diseases may exhibit upregulation of Helios in the absence of Foxp3 expression. Conversely, as a small percentage (10-15%) of Foxp3^+^Helios^-^ T cells can be found in normal PBMC, it remains possible that under certain inflammatory conditions *in vivo*, one might observe an expansion of this population. This population must be distinguished from effector T cells by their failure to produce IL-2.

In summary, our *in vivo* studies both in humanized mice and in patients demonstrate that Foxp3 expression is not upregulated in human T_conv_ when activated under a variety of inflammatory conditions. Thus, Foxp3 expression alone can be used as a marker for bona fide T_reg_ *in vivo*. However, as we did not perform an exhaustive study of a wide spectrum of autoimmune and inflammatory diseases, we would suggest that staining for Helios be used in conjunction with Foxp3 staining to confirm that the Foxp3^+^ T cells are true T_reg_. In addition, the combination of Foxp3 and Helios should be mandatory for quantification of T_reg_ that have been expanded *in vitro* for use in cellular biotherapy or for production of CAR-T_reg_ (27).

## Data availability statement

The original contributions presented in the study are included in the article/**Supplementary Material**, further inquiries can be directed to the corresponding author.

## Ethics statement

The studies involving human participants were reviewed and approved by Institutional Review Boards of NIAMS and NCI. The patients/participants provided their written informed consent sto participate in this study.

## Author contributions

Contribution: MB and ES designed the study and wrote the manuscript; LM and MJ performed experiments and wrote the manuscript; AG, CF, CK provided patient samples and reviewed the manuscript; CO analyze data and wrote manuscript. All authors contributed to the article and approved the submitted version. 

## References

[1] Fontenot JD Gavin MA Rudensky AY. Foxp3 programs the development and function of CD4+CD25+ regulatory T cells. Nat Immunol (2003) 4:330–6. doi: 10.1038/ni904 12612578

[2] Sakaguchi S Mikami N Wing JB Tanaka A Ichiyama K Ohkura N. Regulatory T cells and human disease. Ann Rev Immunol (2020) 38:541–66. doi: 10.1146/annurev-immunol-042718-041717 32017635

[3] Thornton AM Korty PE Tran DQ Wohlfert EA Murray PE Belkaid Y. Expression of Helios, an ikaros transcription factor family member, differentiates thymic-derived from peripherally induced Foxp3^+^ T regulatory cells. J Immunol (2010) 184:3433–344. doi: 10.4049/jimmunol.0904028 PMC3725574 20181882

[4] Sebastian M Lopez-Ocasio M Metidji A Rieder SA Shevach EM. Thornton AM Helios controls a limited subset of regulatory T cell functions. J Immunol (2016) 196:144–55. doi: 10.4049/jimmunol.1501704 PMC4685018 26582951

[5] Thornton AM Lu J Korty PE Kim YC Martens C Sung PD. Helios + and Helios – treg subpopulations are phenotypically and functionally distinct and express dissimilar TCR repertoires. Eur J Immunol (2019) 49:398–412. doi: 10.1002/eji.201847935 30620397 PMC6402968

[6] Miyao T Floess S Setoguchi R Luche H Fehling HJ Waldmann H. Plasticity of foxp3+ T cells reflects promiscuous Foxp3 expression in conventional T cells but not reprogramming of regulatory T cells. Immunity (2012) 36:262–75. doi: 10.1016/j.immuni.2011.12.012 22326580

[7] Liu W Putnam AL Zhou XY Szot GL Lee MR Zhu S. CD127 expression inversely correlates with FoxP3 and suppressive function of human CD4^+^ T reg cells. J Exp Med (2006) 203:1701–11. doi: 10.1084/jem.20060772 PMC2118339 16818678

[8] Sedikki N Santner-Nanan B Martinson J Zaunders J Sasson S Landay A. Expression of interleukin (IL)-2 and IL-7 receptors discriminates between human regulatory and activated T cells. J Exp Med (2006) 203:1693–700. doi: 10.1084/jem.20060468 PMC2118333 16818676

[9] Allan SE Crome SQ Crellin NK Passerini L Steiner TS Bacchetta R. Activation-induced Foxp3 in human T effector cells does not suppress proliferation or cytokine production. Int Immunol (2007) 19:345–54. doi: 10.1093/intimm/dxm014 17329235

[10] Kmieciak M Gowda M Graham L Godder K Bear HD Marincola FM. Human T cells express CD25 and Foxp3 upon activation and exhibit effector/memory phenotypes without any regulatory/suppressor function. J Trans Med (2009) 7. doi: 10.1186/1479-5876-7-89 PMC2770477 19849846

[11] Tran DQ Ramsey H Shevach EM. Induction of foxp3 expression in naive human CD4+Foxp3– T cells by T-cell receptor stimulation is transforming growth factor-β–dependent but does not confer a regulatory phenotype. Blood (2007) 110:2983–90. doi: 10.1182/blood-2007-06-094656 PMC2018674 17644734

[12] Wang J Ioan-Facsinay A van der Voort EI Huizinga TWJ Toes REM. Transient expression of FOXP3 in human activated nonregulatory CD4+ T cells. Eur J Immunol (2007) 37:129–38. doi: 10.1002/eji.200636435 17154262

[13] King MA Covassin L Brehm MA Racki W Pearson T Leif J. Human peripheral blood leucocyte non-obese diabetic-severe combined immunodeficiency interleukin-2 receptor gamma chain gene mouse model of XENOGENEIC graft-*versus*-host-like disease and the role of host major histocompatibility complex. Clin Exp Immunol (2009) 157:104–18. doi: 10.1111/j.1365-2249.2009.03933.x PMC2710598 19659776

[14] Conran N Belcher JD. Inflammation in sickle cell disease. Clin Hemorheol Microcirc (2018) 68:263–99. doi: 10.3233/CH-189012 PMC6314308 29614637

[15] Liu C Martins AJ Lau WW Rachmaninoff N Chen JG Imberti L. Time-resolved systems immunology reveals a late juncture linked to fatal covid-19. Cell (2021) 184:1836–57. doi: 10.1016/j.cell.2021.02.018 PMC7874909 33713619

[16] Stuart T Butler A Hoffman P Hafemeister C Papalexi E Mauck WM. Comprehensive integration of single-cell data. Cell (2019) 177:1888–902. doi: 10.1016/j.cell.2019.05.031 PMC6687398 31178118

[17] Becht E McInnes L Healy J Duterte CA Kwok IWH Ng LG. Dimensionality reduction for visualizing single-cell data using UMAP. Nat Biotechnol (2018) 37:38–44. doi: 10.1038/nbt.4314 30531897

[18] Finak G McDavid A Yajima M Deng JY Gersuk V Shalek AK. MAST: a flexible statistical framework for assessing transcriptional changes and characterizing heterogeneity in single-cell RNA sequencing data. Genome Biol (2015) 16:278. doi: 10.1186/s13059-015-0844-5 26653891 PMC4676162

[19] Delorey TM Ziegler CG Heimberg G Normand R Yang Y Segerstolpe A. Covid-19 tissue atlases reveal SARS-COV-2 pathology and cellular targets. Nature (2021) 59:107–13. doi: 10.1038/s41586-021-03570-8 PMC8919505 33915569

[20] Suárez-Fueyo A Bradley SJ Tsokos GC. T Cells in systemic lupus erythematosus. Curr Op Immunol (2016) 43:32–8. doi: 10.1016/j.coi.2016.09.001 PMC5125867 27636649

[21] Moulton VR Tsokos GC. T Cell signaling abnormalities contribute to aberrant immune cell function and autoimmunity. J Clin Invest (2015) 125:2220–7. doi: 10.1172/JCI78087 PMC4497749 25961450

[22] Ragab D Salah Eldin H Taeimah M Khattab R Salem R. The COVID-19 cytokine storm; what we know so far. Front Immunol (2020) 11:1446. doi: 10.3389/fimmu.2020.01446 32612617 PMC7308649

[23] Brown ME Peters LD Hanbali SR Arnoletti JM Sachs LK Nguyen KQ. Human CD4+CD25+CD226- tregs demonstrate increased purity, lineage stability, and suppressive capacity versus CD4+CD25+CD127lo/- tregs for adoptive cell therapy. Front Immunol (2022) 13:873560. doi: 10.3389/fimmu.2022.873560 35693814 PMC9178079

[24] Kim H-J Barnitz RA Kreslavsky T Brown FD Moffett H Lemieux ME. Stable inhibitory activity of regulatory T cells requires the transcription factor Helios. Science (2015) 350:334–9. doi: 10.1126/science.aad0616 PMC4627635 26472910

[25] Kim YC Bhairavabhotla R Yoon J Golding A Thornton AM Tran DQ. Oligodeoxynucleotides stabilize Helios-expressing Foxp3+ human T regulatory cells during *in vitro* expansion. Blood (2012) 119:2810–8. doi: 10.1182/blood-2011-09-377895 PMC3327459 22294730

[26] Lam AJ Uday P Gillies JK Levings MK. Helios Is a marker, not a driver, of human treg stability. Eur J Immunol (2022) 52:75–84. doi: 10.1002/eji.202149318 34561855

[27] Boardman DA Levings MK. Emerging strategies for treating autoimmune disorders with genetically modified treg cells. J Allergy Clin Immunol (2022) 749:1–11. doi: 10.1016/j.jaci.2021.11.007 34998473

