# Supplementary material for: Co-expression of Foxp3 and Helios facilitates the identification of human T regulatory cells in health and disease
Source: Front Immunol. 2023 Jun 7;14:1114780. doi: 10.3389/fimmu.2023.1114780 (PMC10282999; doi:10.3389/fimmu.2023.1114780)
Supplement: Supplementary file 1 [file DataSheet_1.pdf]

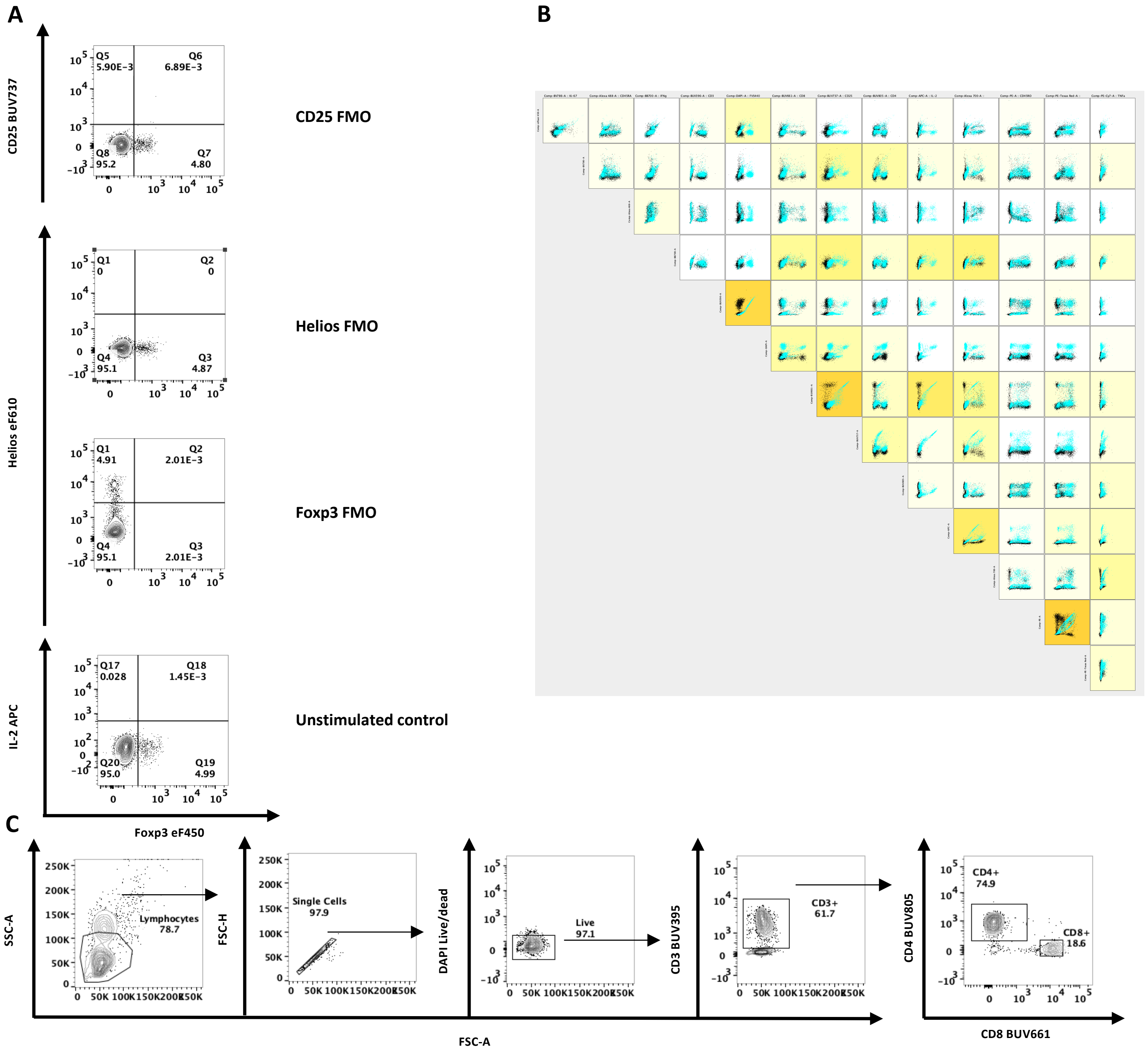

Supplementary Figure 1. Controls used for gating and analyzing flow cytometry data. (A) FMO control plots for gating of CD25, Helios and Foxp3. Flow cytometry stainings are shown along with a plot from a sample that was not stimulated with PMA + Ionomycin + protein transport inhibitor. (B) Example compensation matrix NxN plot with compensated (in black) and an uncompensated overlay (in blue) is also shown. (C) Example hierarchical gating strategy used for the experiments shown in this paper. hCD45 was added for experiments that used NSG mice.

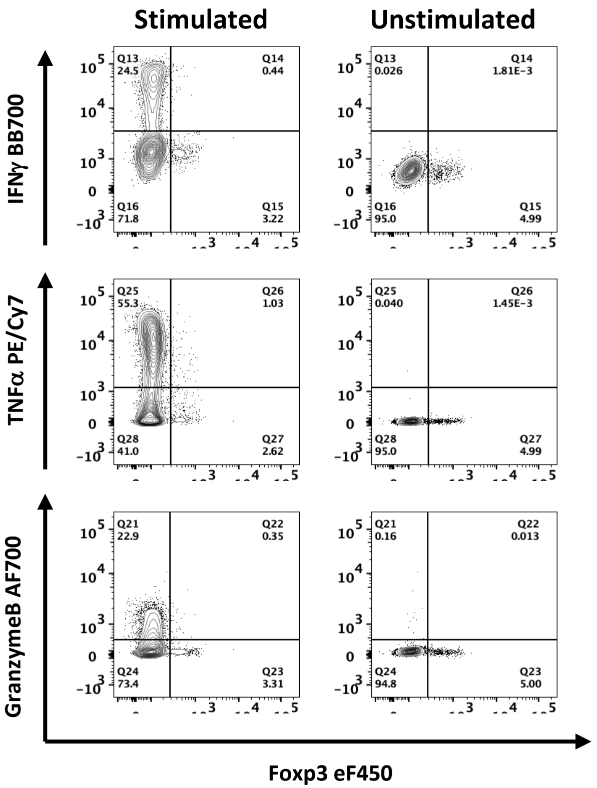

Supplementary Figure 2. Activation does not induce cytokine production in Foxp3$^+$Helios$^+$CD4$^+$ T cells. PBMCs from healthy donors were left unstimulated or cultured for 72h with anti-CD3/anti-CD28 coated beads and IL-2. All samples were then stimulated with PMA/ionomycin for 2 hours and stained for IFNγ, TNFα, and Granzyme B. One representative sample of 3 is shown. Gated on live, CD3+, CD4+ lymphocytes.

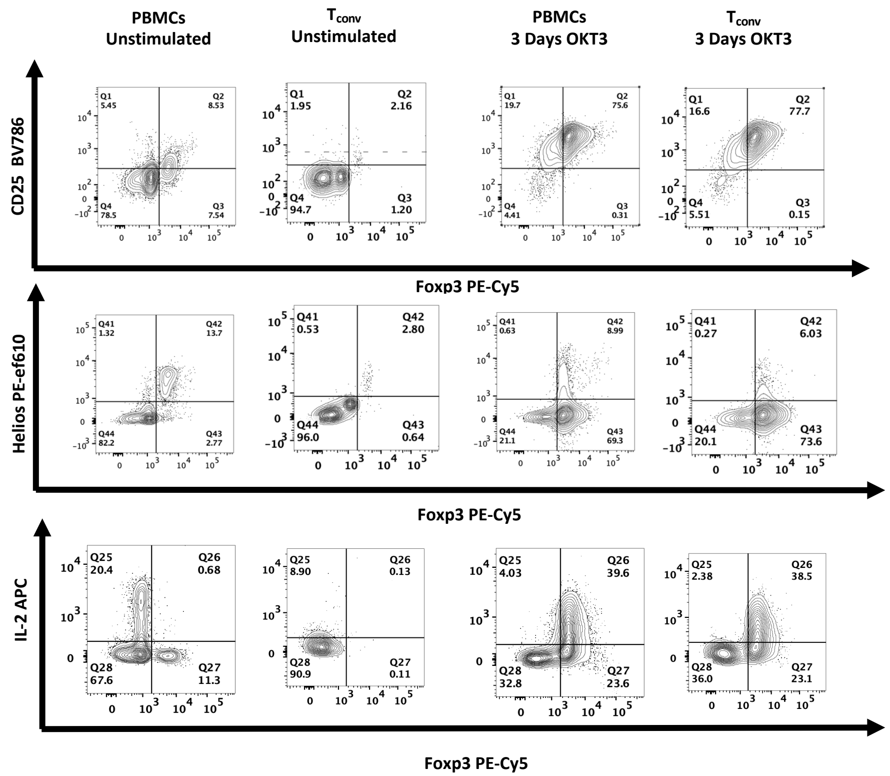

Supplementary Figure 3. Activation of sorted CD4$^+$CD25$^-$CD127^{hi} T$_{conv}$ *in vitro* induces upregulation of Foxp3, but not Helios. Sorted CD4$^+$CD25$^-$CD127^{hi} T cells (T$_{conv}$) were stained with CTV proliferation dye, mixed with unlabeled autologous PBMCs from healthy donors and stimulated with anti-CD3 (OKT3). After 3 days, cells were gated on CTV$^+$ purified CD4$^+$ T$_{conv}$ and CTV$^-$ PBMC and stained for Foxp3, Helios, and IL-2 as in Figure 1.

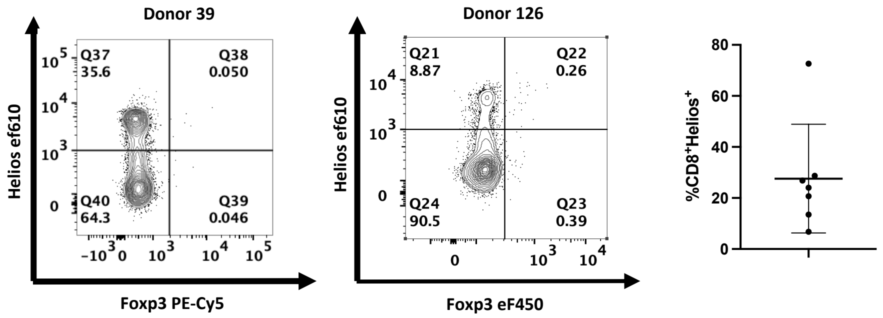

Supplementary Figure 4. Resting CD8⁺ T cells express Helios. (A) Human PBMCs were analyzed for expression of Helios in CD8⁺ T cells. Two representative donors are shown. (B) Summary of the percentage of CD8⁺ Helios⁺ cells from multiple donors (*n=7*).

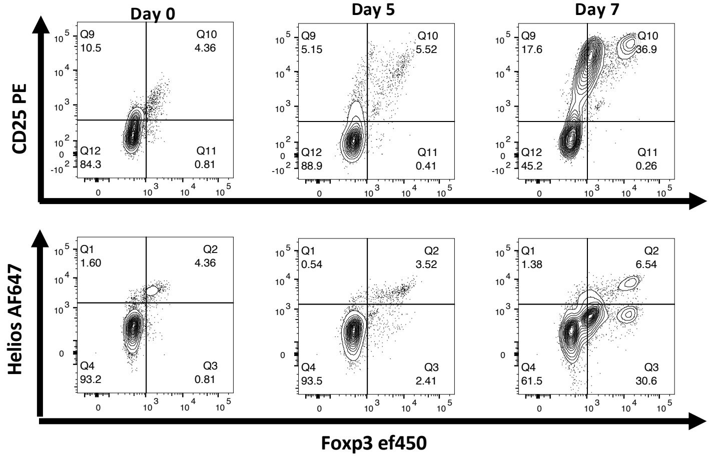

Supplementary Figure 5. Activation of Human PBMCs *in vitro* in a xeno-MLR. hPBMCs were cultured with splenocytes from NSG mice at a ratio of 1:1 for a total of 1x10^6 cells. Gated CD4$^+$ T cells were analyzed on days 5 and 7 for the indicated activation markers.

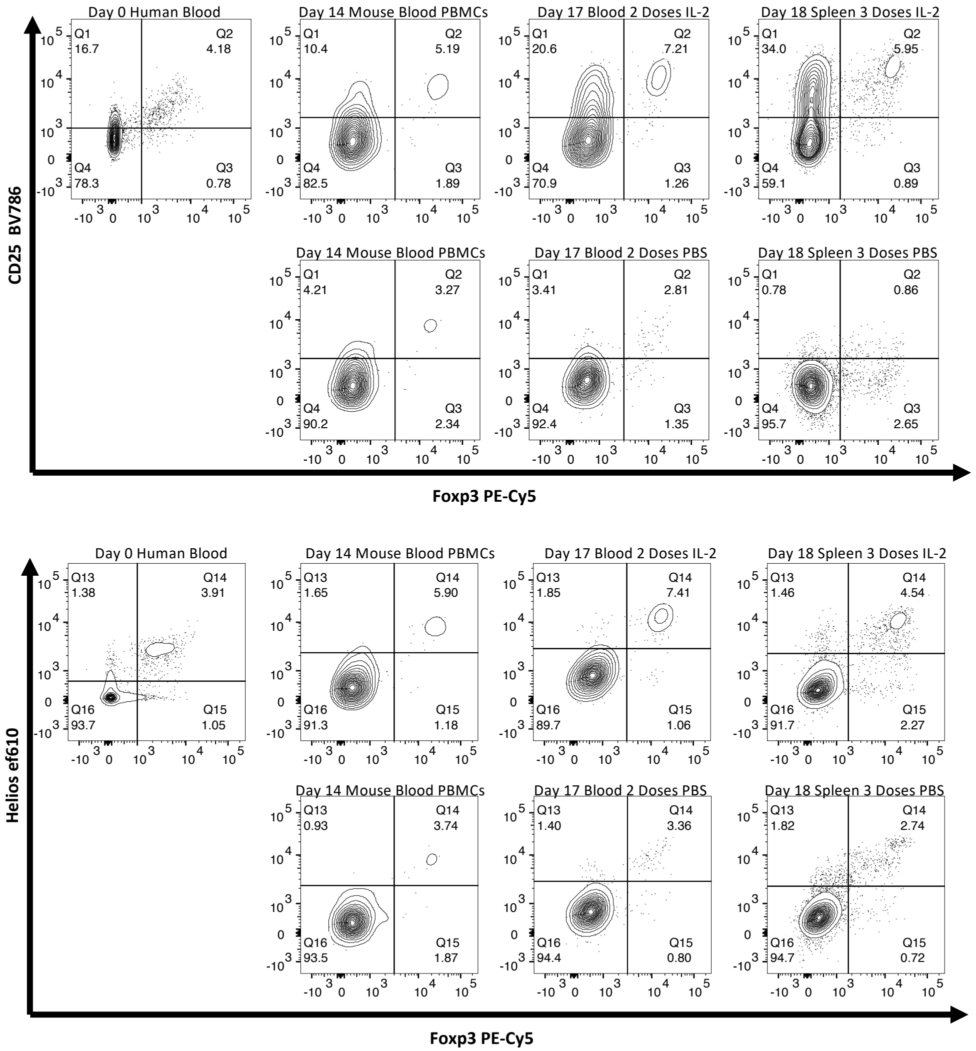

Supplementary Figure 6. Activation of human PBMCs *in vivo* with IL-2. hPBMCs were engrafted into NSG mice and mice were injected with 3 doses of hIL-2 (2 x 10^5 IU) or with PBS (*n=3, both groups*). Blood from day 14, before IL-2 injections began, day 17, after two doses of IL-2, and splenocytes from day 18 were gated on $CD4^+$ T cells and analyzed to detect CD25, Foxp3, and Helios expression. Results are representative of 2 independent experiments.

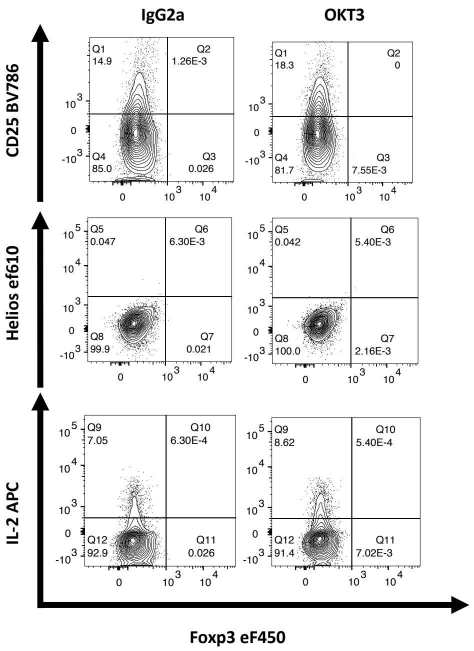

Supplementary Figure 7. Activation of Human CD4^{+} T$_{conv}$ *in vivo* with OKT3. NSG mice were engrafted with hPBMCs and injected with OKT3 (125 μg/mouse, *n=5*) or IgG2a (*n*=6) intravenously on day 14. After 12 hours, spleens were harvested and analyzed by flow cytometry. Representative data is shown from one of two independent experiments.

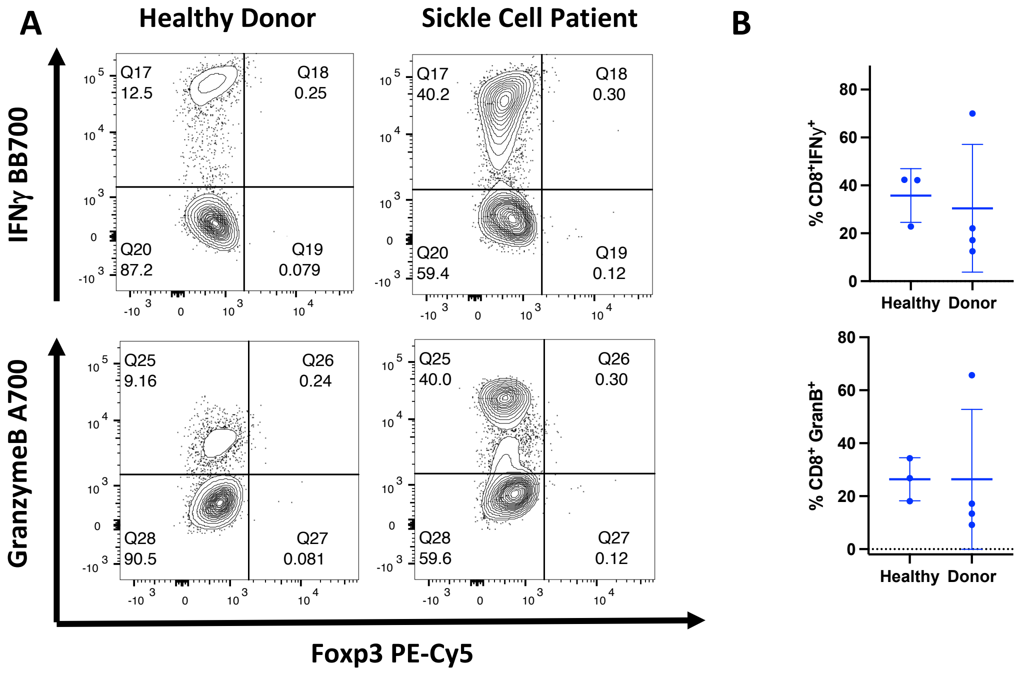

Supplementary Figure 8. Cytokine production by CD8$^+$ T cells from SCD patients. PBMCs from patients with SCD and healthy donors were analyzed by flow cytometry. The IFNγ^+CD8$^+$ and GranzymeB$^+$CD8$^+$ populations from one representative donor of 4 is shown. (B) Summary data on cytokine expression in patients compared with healthy donors (all comparisons are ns).

Regression_coefficient	Std. Error	pvalue	Variable
0.097585352	0.0139586	3.06E-12	IL2RA
0.217796552	0.0391603	2.8E-08	GenderMale
0.063293575	0.0130631	1.3E-06	IL32
0.003870869	0.0008434	4.54E-06	Age
-0.261255242	0.0617341	2.35E-05	BatchB3
0.054503112	0.0140424	0.000105	CORO1A
-0.234366292	0.0608395	0.000118	BatchB2
0.043614482	0.0139839	0.001825	CD3D
0.04218903	0.0143784	0.003358	LCK
0.05136436	0.0187158	0.006081	BCL11B
0.057626063	0.0213734	0.007036	RASGRP1
0.066379931	0.024692	0.007203	TNFRSF18
0.132120335	0.0513335	0.010086	CXCR6
0.031765742	0.0134662	0.018363	MATR3
0.03384325	0.0147142	0.021484	PTPRCAP
0.05501495	0.0239362	0.021577	SLAMF1
0.031177318	0.0143843	0.030243	CD27
0.035186918	0.0167275	0.035464	CCR6
0.026706687	0.0135113	0.048135	CD247
0.029647461	0.0153936	0.054161	BATF
0.025943263	0.0140021	0.063964	ACAP1
0.021050644	0.0131676	0.109951	STK17B
-0.022201795	0.0153802	0.148929	CD28
0.040615413	0.0285364	0.15471	MAF
-0.032423568	0.0240575	0.177794	PBX4
-0.072588821	0.0539885	0.178835	PDCD1
-0.019779161	0.0154068	0.199269	FYN
-0.015248544	0.012415	0.219412	LTB
-0.016249112	0.0132786	0.221115	SPOCK2
-0.470027773	0.3912502	0.229668	IL2
-0.018382117	0.0162764	0.258789	ICOS
0.015905759	0.0140906	0.259022	TIGIT
-0.044429594	0.0395807	0.261697	CD40LG
0.015140718	0.0138322	0.273739	SKAP1
-0.021017558	0.0192042	0.273816	GIMAP5
-0.01761726	0.0166285	0.289438	CD96
0.043085591	0.0427811	0.313923	SNX29P2
0.015148184	0.0152357	0.320144	`PRKCQ-AS1`
0.014692812	0.016358	0.369115	CD6
0.013917603	0.017224	0.419107	WASH4P
-0.011492901	0.0143658	0.423736	GIMAP7
0.010549204	0.0137659	0.443514	IL2RG
-0.10395007	0.1423192	0.465177	B3GALT2
0.015312239	0.0213535	0.473354	TNFRSF25
0.013345143	0.0199244	0.503021	PTPN7
0.032841589	0.0560734	0.558108	HC_non_Treg
0.013526375	0.0236963	0.568143	MIR142
0.008111408	0.0143539	0.572028	ITK
0.007830067	0.014101	0.578722	TRAF3IP3
-0.006413032	0.0119007	0.589992	IL7R
0.008296618	0.0184853	0.653578	IKZF2
-0.006132192	0.0138584	0.658152	CTLA4
-0.005941477	0.0136531	0.663453	ETS1
0.022982805	0.0549216	0.675623	COVID_non_Treg
0.00782852	0.0196341	0.690115	IL2RB
0.007465541	0.0204729	0.715384	SIT1
-0.006540682	0.0203398	0.747791	TNFRSF4
-0.005952837	0.0189489	0.753418	RUNX3
0.003755184	0.0139522	0.787828	CD3G
0.014665721	0.0591537	0.804201	COVID_Treg
0.016750345	0.0748525	0.822939	TMIGD2
-0.004281485	0.0200759	0.831128	SIRPG
0.002968433	0.0168525	0.860189	CD3E
-0.001741428	0.0138311	0.89981	CAMK4
0.00150418	0.0132029	0.909299	CD7
0.001325841	0.0246945	0.957184	ZC3H12D
-0.001126017	0.0234137	0.961644	PTPN22
-0.000249755	0.0130882	0.984776	CD2

Supplementary Table 1. Linear regression analysis results in which FOXP3 expression in cluster 6 is modeled as a function of 68 variables. Regressions coefficients and p-values associated with the 68 potential predictors of FOXP3 expression, including the expression levels of the Treg signature genes, age, gender, batch, and the cell population identity defined by the combined patient status (Healthy Control/COVID) and cell type (T_{reg}/non-Treg).
